# The mycobacterial selenocysteine machinery: presence and expression

**DOI:** 10.1080/15476286.2026.2712054

**Published:** 2026-07-30

**Authors:** Phani Rama Krishna Behra, Malavika Ramesh, B. M. Fredrik Pettersson, Leif A. Kirsebom

**Affiliations:** Department of Cell and Molecular Biology, Uppsala University, Uppsala, Sweden

**Keywords:** Mycobacteria, selenocysteine, selenoprotein, tRNA-Sec (selC), selenocysteine inserstion sequence (SECIS)

## Abstract

The *Mycobacterium* genus includes more than 190 species that occupies diverse ecological niches. Some are non-pathogenic and environmental, whereas others cause severe diseases both in humans and animals, *e.g*. tuberculosis (TB) and leprosy. Selenocysteine (Sec) is present in all three domains of life. Here we report the presence of the Sec-machinery (*selA*, *selB*, *selD* and tRNA^Sec^) genes and selenoprotein formate dehydrogenase (FDH) genes in roughly 40% of 244 mycobacterial genomes. Their presence is distributed evenly among slow and rapid-growing mycobacteria and our data indicate that they were acquired through horizontal gene transfer. Some mycobacteria however lost these genes during the evolution of the genus. We provide RNA-Seq data showing transcript levels of the Sec-machinery and FDH genes in different mycobacteria grown under different conditions. Finally, we suggest that the tRNA^Sec^ gene (*selC*), positioned immediately upstream of *selA-selB*, is involved in the regulation of the expression of *selA-selB*. Together our data expand our understanding of selenocysteine metabolism and its evolution within the *Mycobacterium* genus.

## Introduction

Selenium (Se) was first identified in 1818 by the Swedish chemist, Jöns Jacob Berzelius. For many organisms, selenium is an essential trace element. For humans it is vital and it can influence human health. In 1954, selenium was shown to have a role in microbial metabolism ([1,2] and Refs therein). Among bacteria, selenium exists in three forms: (i) as selenocysteine, Sec (the 21st amino acid) in specific proteins, which are also present in all domains of life, (ii) as a non-covalently bound cofactor and (iii) as a modification, 5-methylaminomethyl-2-selenouridine, in certain tRNAs (tRNA^Glu^, tRNA^Lys^ and tRNA^Gln^) ([3,4] and Refs therein). The insertion of Sec into a protein requires an inframe UGA codon, which is generally used as a translational stop codon, and an RNA structural element present in the mRNA, referred to as the selenocysteine insertion sequence (SECIS). The size of SECIS is roughly 60 nucleotides and it is folded into a stem loop structure. In bacteria, it is positioned immediately downstream of the UGA codon [5,6]. The UGA codon is read by a specific tRNA, tRNA^Sec^(UCA), and its gene is referred to as *selC* [7]. First, seryl-tRNA-synthetase (*serS*) charges tRNA^Sec^(UCA) with serine generating Ser-tRNA^Sec^(UCA). The serine is then converted to Sec-tRNA^Sec^ by selenocysteine synthase (s*elA*) and selenophosphate (SePO^3-^_3_), which provides selenium. SePO^3-^_3_ is generated from Se and ATP, and this reaction is catalysed by selenophosphate synthetase (*selD*). The Sec-tRNA^Sec^, forms a complex with the specific elongation factor SelB (*selB*) and GTP. This complex brings Sec-tRNA^Sec^ to the A site on the ribosome that exposes UGA with the SECIS element in its close proximity, which interacts with SelB [8]. The outcome is incorporation of Sec, and proteins having Sec are referred to as selenoproteins. The majority of known selenoproteins are enzymes involved in various cellular functions such as redox reactions [4,9]. In bacteria, SelA, SelB, tRNA^Sec^ (*selC*) and SelD are referred to as the Sec incorporating machinery [3,5,10]. In addition, SelU (gene *selU* or *ybbB*) is involved in mnm^5^Se^2^U synthesis (modification) on certain tRNAs (see above) [9]. The most abundant translational stop codon among Actinobacteria and mycobacteria is UGA [11]. Hence, standard annotation systems often fail to identify/predict selenoproteins. Finding selenoproteins can therefore be based on the presence of the Sec-machinery and the presence of SECIS in close proximity to the codon UGA in the mRNA.

Initially, selenoproteins were identified in *Escherichia coli* and in *Clostridium* species by using genetic approaches [3,7,12]. With the advancement of genome sequencing, and the availability of a growing number of bacterial genomes, offer new possibilities to identify the presence of the Sec-machinery and selenoprotein genes [1,13–16]. Comparative genomics has been used to identify the presence of the Sec-machinery and selenoproteins in roughly 20% of sequenced bacterial genomes. Among these, 56 mycobacteria have been reported to have Sec-machinery genes [9,17–25]. We recently reported the presence of the tRNA^Sec^ gene (*selC*) in 102 mycobacterial genomes [26]. Having access to the majority of the mycobacterial genomes as well as several genomes of specific mycobacterial strains [26,27] gave us the incentive to map the presence of the Sec-machinery and selenoprotein genes among mycobacteria. This would provide insight and expand our understanding of the selenocysteine metabolism, and the evolution and distribution of the Sec-machinery within the *Mycobacterium* genus.

We applied a comparative genomics approach using 244 mycobacterial genomes, which included 192 named species representing the *Mycobacterium* genus. We identified genes constituting the Sec-machinery (*selA*, *selB*, *selC* and *selD*) in approximately 40% of the 244 genomes, while no homolog of *selU* could be detected in any of the analysed genomes. Our data further suggest that the Sec-machinery and selenoprotein (formate dehydrogenase, FDH) genes have been acquired through horizontal gene transfer. We also provide RNA-Seq data for selected slow (SGM) and rapid (RGM) growing mycobacteria showing the transcript levels for the Sec-machinery genes and the predicted sole selenoprotein FDH gene in response to growth conditions and exposure to different stress conditions relevant to mycobacterial infections.

## Materials and Methods

### Identifying the selenocysteine tRNA^Sec^ and SECIS

To identify the tRNA^Sec^ gene (*selC*) in 244 mycobacteria, we used the tools tRNAScanSE [28,29], Secmarker [22], and the Rfam database [30]. The presence of tRNA^Sec^ genes were searched for using the programme tRNAScanSE with default settings. Next, using the specialized tool, Secmarker v0.4, the tRNA^Sec^ gene was identified with the default settings. Also, by using the Rfam database v13.0 along with the infernal; cmsearch command setting the parameter (threshold cut-off) ‘−T’ 45 and run with the default settings identified both ‘tRNA^Sec^’ and ‘SECIS’. Also, SECIS was analysed using the bSECISearch tool [31]. Finally, we combined these methods and verified the presence of tRNA^Sec^ and SECIS through manual inspection.

### Selenocysteine, Sec, machinery and selenoproteins

To identify Sec protein-coding genes, we used the selenoprofiles v3.6 tool [23], which was designed mainly to identify the selenocysteine machinery in complete and draft genomes. This pipeline consists of three steps, mainly BLAST search, exonerate and genewise and the user can adjust parameters for each profile or all profiles. In our analysis, we set the parameters as per the manual suggested for bacterial genome sequences with default settings and minimum cut-off of 60% identity and query coverage.

All 244 genome sequences were previously annotated using the PROKKA v1.11 annotation pipeline [26] and searched for open reading frames of Sec-related genes. Data were extracted based on GFF files, with the text search ‘Seleno or *selA* or *selB* or *selD* or selenocysteine’. To confirm missing gene sequences and identify annotation omitted genes, we used BLAST tool blastN search with the cut-off of percentage identity as 70% and query coverage HSP as 60%. We combined these methods and together with manual inspection we identified the presence and absence of Sec-related genes.

### Sequence alignment

The multiple sequence alignments were performed by using the clustalw aligner [32] and the sequences were visualized with the Jalview tool [33].

### Core gene phylogeny

The core gene phylogenetic tree was obtained from Behra et al. [26]. In brief, 387 orthogroups were identified among 244 mycobacterial genome sequences using the SCARAP pipeline. The corresponding protein sequences were concatenated and aligned using the tool MAFFT v7.407 [34]. Phylogenetic tree construction was done using the tool FastTree v2.1.10 [35], by default setting, which uses protein sequence (Jukes canter model). The phylogenetic tree was generated using the tool ITOLtree v3 [36].

### RNA extraction and RNA sequencing

RNA-Seq data (two biological replicates) for selected slow-growing mycobacteria, SGM, including four *M. marinum* strains (CCUG20998, 1218 R, 1218S and M) and *M. montefiorense* DSM44602, and three rapid growing mycobacteria, RGM (*M. elephantis* DSM44368, *M. mageritense* DSM44476, and *M. monacense* DSM44395/pD5), were analysed.

These mycobacteria were grown, either in 7H9 liquid media or on 7H10 plates, at recommended temperatures (see Table S1; note, no information about the Se source in these media are available) and total RNA was extracted from exponential and stationary phase cells as reported elsewhere [37,38]. For growth in 7H9 liquid media and exposure to the different stress conditions and oxygen depletion see Ref [37]. To achieve oxygen depletion, we followed the Wayne model [39,40], and the times for oxygen depletion were, for *M. marinum* CCUG20998 – 0 hours (control), 9 hours, 1 day, 3 days, 6 days and 12 days – and for *M. monacense* DSM44395/pD5 – 0 hours (control), 8 hours, 1 day, 6 days and 12 days. In this context, we included and analysed our previously reported RNA-Seq data for the *M. marinum* CCUG20998 strain in response to various stressors compared to the control sample [37]. Since the focus is on the *Mycobacterium* genus, we name the different mycobacteria as, *e.g*. *M. marinum*, throughout the text.

The respective RNA-Seq short read data were mapped to the respective reference genome using the pipeline Tophat v 2.0.13 [41] and aligned with bowtie2 v2-2–4 [42]. Read counts were generated using the HTSeq v0.9.1 [43] followed by normalization and differential expression analysis using the Deseq2 R package [44], which gives the statistical significance expressed as P+adj values.

To quantify mRNA abundance and compare the expression of different species, we calculated transcripts per million (TPM) values using the TPMCalculator tool [45]. The TPM values were plotted using the R programme v3.2.2 [46] in conjunction with the ggplot2 R package [47].

### Visualization of data

Gene synteny plot representing the selenocysteine chromosomal loci in different species were plotted using the genoPlotR v0.8.9 R-package [48]. The structural information of the gene tRNA^Sec^, SECIS elements were plotted using the online tool Rd2t software [49]. The bar plots representing the Sec-machinery and FDH (formate dehydrogenase) transcript levels correspond to Deseq2 log_2_-fold values and plotted using the R-package ggplot2 [47].

## Results and discussion

**Figure 1.**
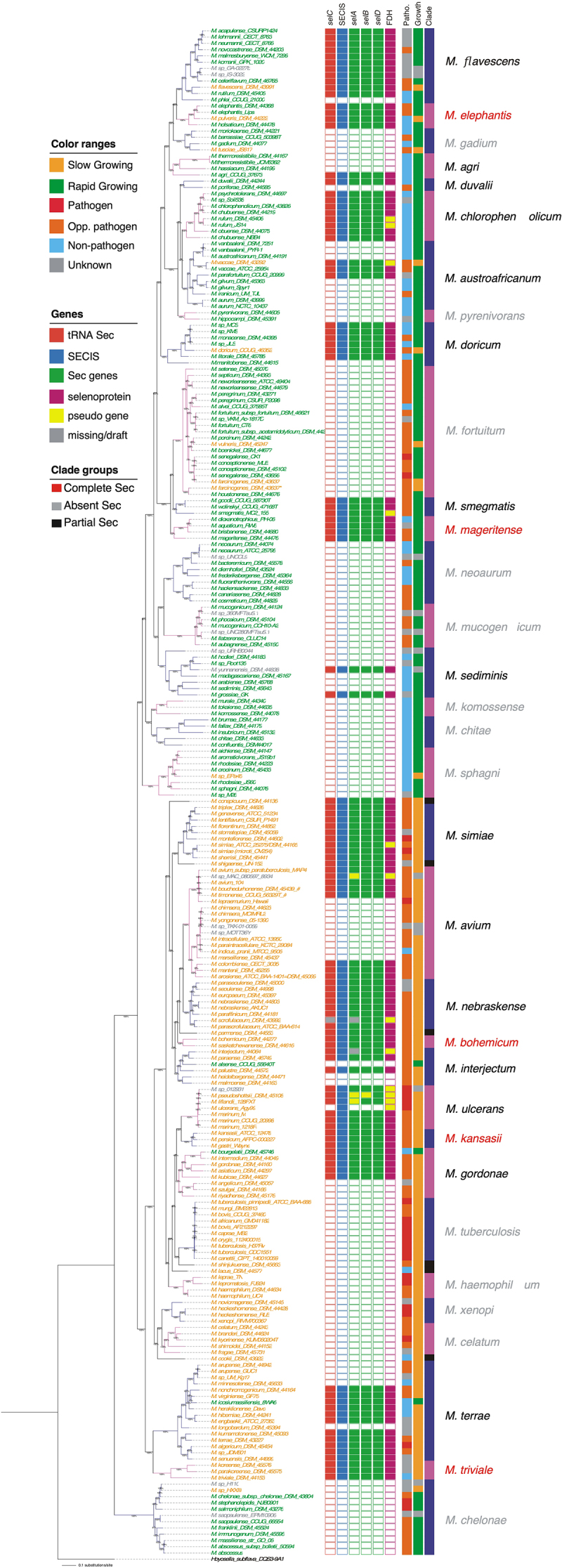
Distribution of selenocysteine genes in mycobacteria.

We recently reported the distribution of tRNA and noncoding RNA genes in 244 mycobacterial genomes and we provided data suggesting the presence of *selC*, tRNA^Sec^(UCA) gene, in 102 mycobacterial genomes (Figure 1) [26]. To get further insight into the Sec-machinery and Sec insertion, we scanned the mycobacterial genomes to identify all Sec-related genes required for a functional Sec-machinery. In this context, the seryl-tRNA synthetase (SerRS) gene (*serS*) is also required for charging of tRNA^Sec^, and we recently reported its presence in all mycobacteria (Table S2a [26]). We also searched for the presence of the SECIS structural motif in order to identify protein candidates carrying Sec, referred to as selenoproteins. The presence of homologous genes in mycobacteria was identified using different approaches as discussed below (see also Materials and Methods).

Heat maps for 244 mycobacteria showing the presence of selenocysteine machinery [*selABD* and *selC* (tRNA^Sec^)] and selenoprotein (FDH) genes based on the combination of predicted results using the tRNAScanSE, Secmarker, Prokka, blastN search and selenoprofile search. The representation of mycobacterial core gene phylogenetic tree (387 genes, 244 species) is retrieved from Behra et al. [26].

### Presence of homologs and structural characteristics of mycobacterial tRNA^Sec^ (*selC*)

In addition to the prediction of the presence of *selC* with tRNAScanSE [26,28,29] we searched the 244 mycobacterial genomes with Secmarker [22] and performed a blastN homology search [50]. These approaches resulted in that *M. grossaie* also has *selC*, in addition to the previously identified mycobacteria. Thus, among the 244 genomes 103 (42%) have *selC* (Table S2a) including 86 named mycobacteria. The distribution of *selC* among mycobacteria was mapped using the 387-core gene phylogenetic tree (Figure 1) [26]. The data showed that the presence of *selC* is spread evenly between SGM and RGM as we recently reported [26]. Homologs of *selC* were also identified in several other actinobacteria but not in all (see Figure S4; and data not shown). Interestingly, the *Hoysella subflava* strain DQS3-9A1 [51,52], which is phyologenetically close to the earliest mycobacterial lineage, the *M. chelonae* clade and used as an outgroup in Figure 1, does not have *selC* (data not shown). This is also the case for the members of the *M. chelonae* clade (Figure 1). This indicate that for the mycobacteria having *selC*, the gene was possibly acquired during the evolution of the *Mycobacterium* genus (see below).

**Figure 2.**
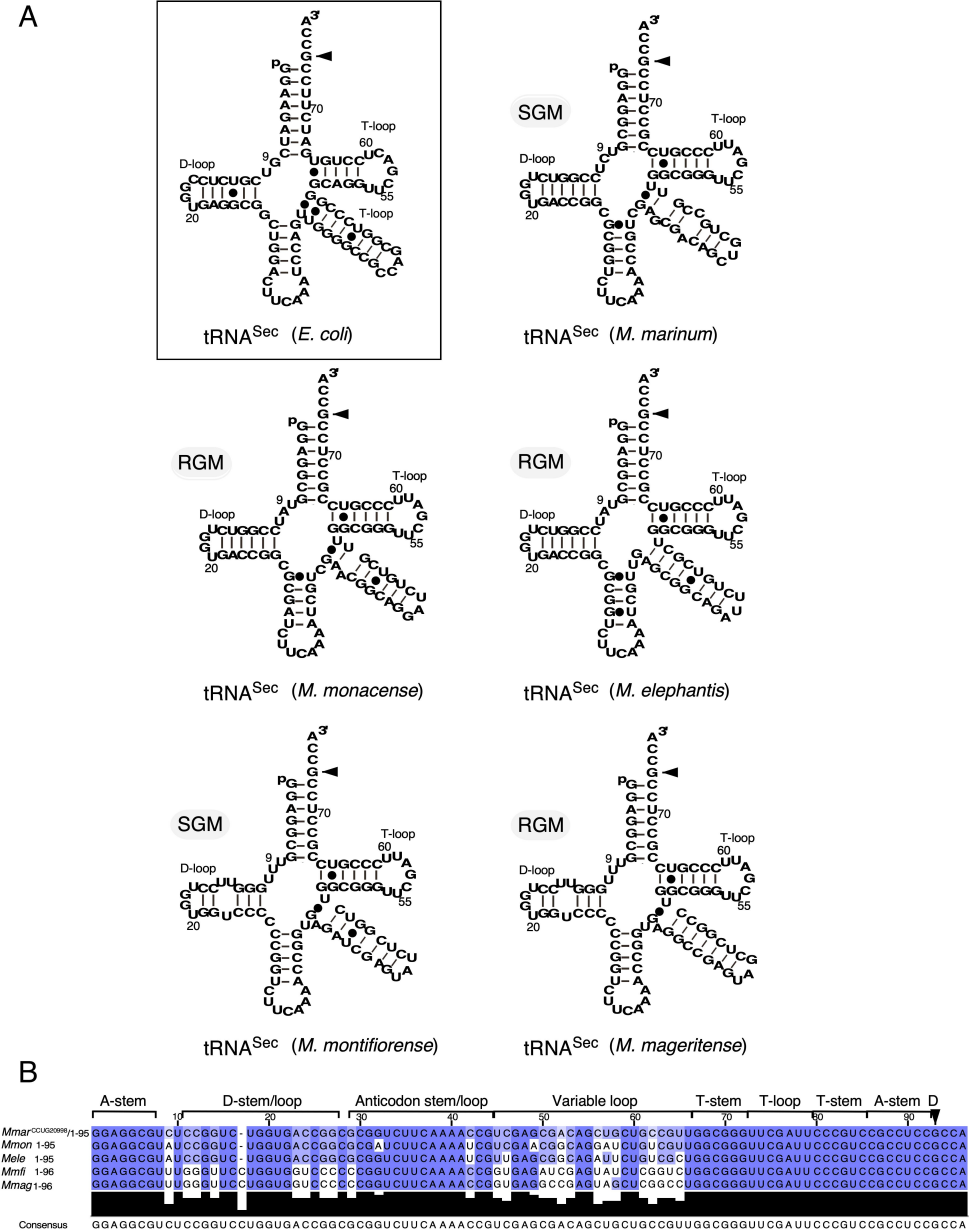
Secondary structures of tRNA^Sec^ and alignment for selected SGM and RGM.

The expression of *selC* is predicted to result in an approximately 95 nucleotides long tRNA^Sec^ (Figures 2 and S1a, b). Compared to the common tRNAs, the variable arm of mycobacterial tRNA^Sec^ is longer, which is in keeping with what has been reported for tRNA^Sec^ in other organisms.

Secondary structure of *E. coli* selenocysteine tRNA and predicted secondary tRNA^Sec^ structures of selected SGM (*M. marinum* and *M. montefiorense*) and RGM (*M. monacense, M. elephantis* and *M. mageritense*). Multiple sequence alignment of *selC* for the selected mycobacteria shown in Figure 2(a).

The ‘acceptor-T-stem’ domains (*i.e*., the combined length of the acceptor- and T-stems measured as the number of base-pairs) in both prokaryotic and eukaryotic tRNA^Sec^ correspond to 13 base-pairs where the acceptor-stem is either 8 or 9 base-pairs long while the number of base-pairs in the T-stem vary between 5 and 4 base-pairs [14,21,22,53,54]. For mycobacteria, the secondary structures suggested that the tRNA^Sec^ acceptor-stem is seven base-pairs long in all mycobacteria while the T-stem is six base-pairs (Figures 2 and S1a-b). Hence, the tRNA^Sec^ ‘acceptor-T-stem’ domain in mycobacteria is 13 base-pairs long consistent with what is known for other organisms.

For mycobacterial tRNA^Sec^, we also detected structural variations in the junction between the acceptor- and D-stem, in the D-stem and in the variable-arm (Figures 2 and S1a, b). Considering the tRNA^Sec^-specific D-stem structure with its six base-pairs is consistent with available structural information for bacterial tRNA^Sec^ [22,53,54] albeit for some mycobacteria the D-stem structure varies (Figures 2 and S1). SelA interacts with the tRNA^Sec^ D-arm structure and its specific structure prevents SelA binding to Ser-tRNA^Ser^ [55,56]. Together, this indicates that mycobacterial tRNA^Sec^ can be selenocysteinylated. However, given the structural variations in the D-arm in some mycobacteria might affect SelA binding and thereby selenocysteinylation. Whether this is the case or not requires further studies. Nevertheless, the sequence variations in the D-arm (and in the junction between the acceptor- and D-stem and in the variable-arm; Figure S1b) might be related to that *selC* was acquired, as discussed above, from different sources and/or due to changes/mutations in these regions during the evolution of the *Mycobacterium* genus (see below Phylogenetic analysis and Supplementary information).

Native tRNA transcripts carry extra nucleotides at the 5’and 3’ ends. These nucleotides are removed by different RNases to generate functional tRNAs [57,58]. The endoribonuclease RNase P is responsible for generating the matured 5’ termini. In general, RNase P cleaves precursor tRNAs between residue −1 and +1 (the residue at the 5’ termini of functional tRNAs) generating 7 bp long amino acid acceptor-stems. The identity of residues N_−1_ and N_+1_ (and a G at + 1 is suggested to act as a guiding nucleotide [57]), the 3’ CCA-motif and the length of the “acceptor-T-stem” domain influence the processing of the tRNA 5’ termini by the endoribonuclease RNase P. Changes in these ‘regions’, *e.g*. absence of the 3’ CCA-motif affect but do not abolish processing. Also, the length of the ’acceptor-T-stem’ domain might vary, as exemplified by that *E. coli* RNase P cleaves the tRNA^Sec^ precursor between residues −2 and −1 generating an 8 bp long amino acid acceptor-stem (Figure 2(a)) ([57,59,60]; also cf. Figure 8 in Ref [61]). Analysis of the mycobacterial tRNA^Sec^ genes revealed that the identity of the residue positioned immediately 5’ of G_+1_ vary and the majority (60%) carry a G at this position (referred to as N_−1_), while the presence of A, C and U are close to 12, 18 and 10%, respectively (Figure S1b and Table S3). Except for *M. conspicium*, *M. gordonae* and *M. grossiae*, all mycobacterial *selC* encodes for the 3’ terminal CCA-motif (Figures 2 and S1b; Table S3). Together, elements responsible to ensure correct RNase P processing are present in mycobacterial tRNA^Sec^. We further note that none of the mycobacteria have the tRNA^His^ guanylyltransferase gene, *tgh*, which adds G to tRNA^His^ in eukaryotes after cleavage by RNase P generating an acceptor-stem with 8 base pairs ([62]; but see Ref [63] for the presence of *tgh* in one *M. phlei* strain).

As discussed above and recently reported, *serS* is present in all mycobacteria (Table S2a) [26]. Based on *E. coli*, the discriminator base, G_+73_ (Figures 2 and S1), is the strongest identity element for SerRS serylation in addition to the long variable-arm and the atypical tRNA^Sec^ secondary structure, which make lesser contributions to the serylation identity. Notable, the charging efficiency is roughly 100-fold lower relative to charging of the serine tRNA isoacceptors. It has been discussed that this is attributed to the atypical tRNA^Sec^ solution conformation relative to serine tRNA [64]. Nevertheless, the structures of mycobacterial tRNA^Sec^ are in keeping with that these tRNAs are recognized by SerRS and charged with serine albeit most likely with lower efficiencies compared to the charging of serine tRNA isoacceptors.

The combined data suggest that structural regions important for mycobacterial tRNA^Sec^ recognition (and processing) were identified and these include: (i) the amino acceptor-stem and the discriminator base that interacts with seryl-tRNA-synthetase, SerRS (aminoacylation of tRNA^Sec^ is dependent on SerRS (*serS*), which catalyses the formation of Ser-tRNA^Sec^ that is subsequently converted into Sec-tRNA^Sec^, see introduction), (ii) the D-loop and D-stem that interact with the N-terminal region of Sec synthase, SelA, and this interaction also discriminates between tRNA^Sec^ and tRNA^Ser^, and (iii) the amino acceptor- and T-stem that interact with the Sec-tRNA^Sec^ specific elongation factor SelB [7,55,56,65,66].

## Identification of Sec-machinery genes – *selA*, *selB* and *selD*

To convert Ser-tRNA^Sec^ to Sec-tRNA^Sec^ requires two proteins SelA and SelD (see above) [10,56,67]. The presence of the corresponding genes *selA* and *selD* were predicted using three approaches: (i) PROKKA annotation followed by extraction of *selA* and *selD* homologs, (ii) homologs were identified using blastN searches with *M. marinum* and *M. smegmatis selA* and *selD* sequences as input queries, and (iii) selenoprofile search for the Sec-machinery and selenoprotein genes based on the Hidden Markov Model, HMM (see also Materials and Methods) [23,50,68].

On the basis of the PROKKA annotation (i), we identified *selA* and *selD* homologs in 94 and 97 mycobacterial genomes, respectively, while the blastN homology search (ii) predicted *selA* and *selD* homologs in 74 and 98 mycobacterial genomes, respectively, and the selenoprofile search (iii) resulted in 91 and 102 mycobacterial genomes having *selA* and *selD* homologs, respectively. Combining the results using these three approaches and by manually verifying the presence of *selA* and *selD* we generated a list of mycobacterial genomes carrying predicted *selA* and *selD* homologs (Figure 1 and Table S2a). We used the same work flow to predict the presence of *selB*, which encodes for the Sec-tRNA^Sec^ specific elongation factor. Among mycobacterial genomes, 97, 65 and 97 were predicted to have *selB* homologs using methods i, ii and iii (see above), respectively, and these are listed in Figure 1 and Table S2a.

Overall, the presence of the tRNA^Sec^ gene among the mycobacterial genomes matches the presence of *selA*, *selB* and *selD*. For one mycobacterium, *M. scrofulaceum*^DSM43992^, we predicted the presence of *selD* and *selB* while we were unable to detect *selA* and *selC*, which is likely due to draft genome status. This is probably also the case for *M. interjectum*^DSM44064^, for which we could not identify *selA*. For *M. ulcerans*^Agy99^, we did not detect the presence of any *selA*, *selB*, *selC* and *selD* homologs while the close relative *M. marinum* (four strains; *M. marinum* 1218S not shown in Figure 1) carries these genes. Interestingly, for *M. liflandii*^128FXT^ and *M. pseudoshottsii*^DSM45108^, which are even closer phylogenetically to *M. ulcerans*^Agy99^ ([26]; note that the *M. ulcerans*^Agy99^ and *M. liflandii*^128FXT^ genomes represent complete genomes [69,70]), we predicted the presence of *selA* and *selB* pseudogenes albeit both carry *selC* (tRNA^Sec^). Data suggest that *M. ulcerans*^Agy99^ diverged from *M. marinum* and the size of its genome has undergone reduction during its evolution [71,72]. The genome sizes for both *M. pseudoshottsii*^DSM45108^ and *M. liflandii*^128FXT^ are also shorter compared to *M. marinum* [26,69,70,73]. This would be consistent with that also these two mycobacteria are subjected to ongoing genome reduction and thereby rationalize the presence of *selA* and *selB* pseudo genes. We recently reported that the genomes of *M. ulcerans* clade members contain high numbers of IS elements and phage sequences. Insertion and shift of IS element location likely leads to the generation of pseudogenes and thereby impact the reductive evolution of *M. ulcerans* clade members [26]. For further insight into the evolution and ecology of *M. ulcerans* (and other mycolactone-producing mycobacteria) we refer to [74,75].

**Figure 3.**
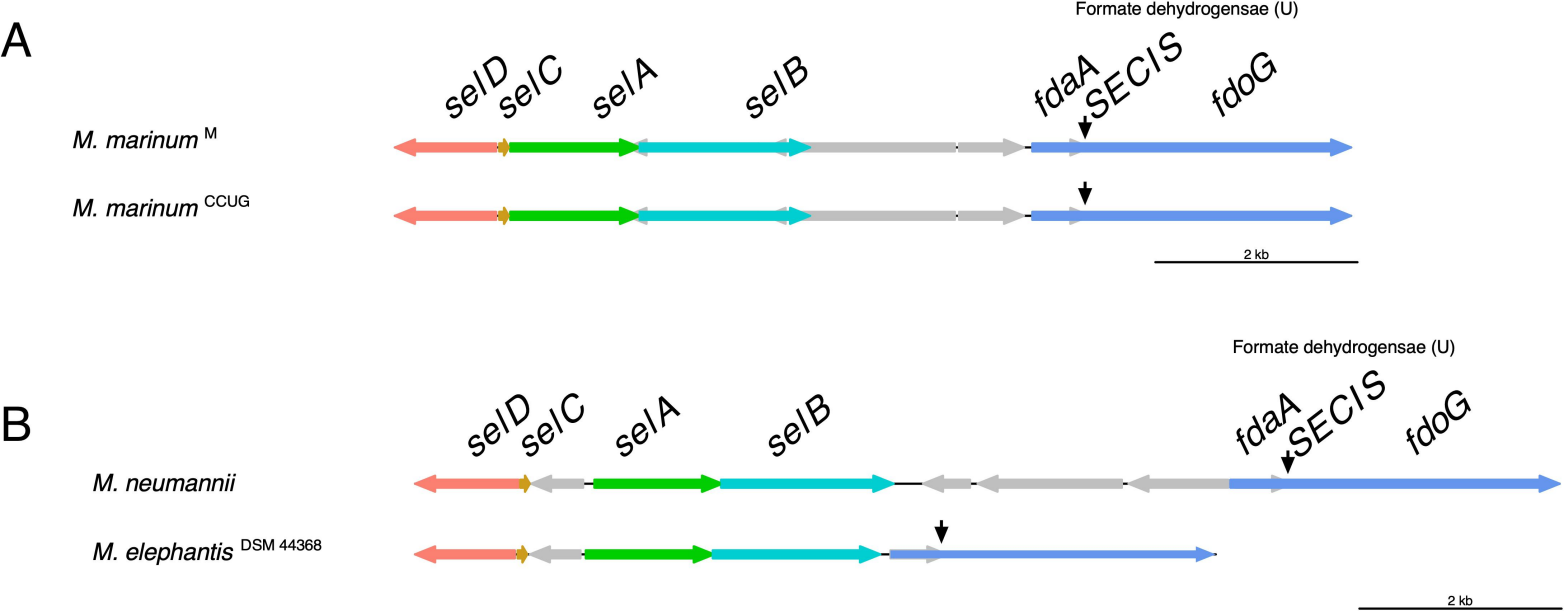
Gene synteny plot showing the selenocysteine genes *selABCD*, positioning of the non-coding RNA SECIS element in *fdhA* (formate dehydrogenase subunit α precursor gene, blue arrow) and *fdoG* (formate dehydrogenase-O major subunit precursor gene), marked with black arrows (see also Figure S2a). (a) Two *M. marinum* strains (SGM) and (b) Two RGM, *M. elephantis* and *M. neumannii* as indicated. The coloured arrows represent the respective *selABCD* homologs.

The gene synteny shows that in mycobacteria *selC* (tRNA^Sec^) is positioned upstream of *selA* and downstream of *selD* (Figures 3(a) and S2a). This is in contrast to *E. coli* where *selC* is located elsewhere on the chromosome and distant to *selA* and *selB* [76]. In some mycobacteria, however, a gene with hypothetical function is positioned between *selC* and *selA*. This is the case for the RGM belonging to the *M. flavescens* and *M. elephantis* clades (Figures 3(b) and S2a). Moreover, *selC*, *selA* and *selB* are transcribed from the same strand while *selD* is transcribed from the opposite strand (Figures 3 and S2a). For those mycobacteria where *selC* is positioned immediately upstream of *selA*, the distance between the tRNA^Sec^ 3’ CCA sequence and the predicted *selA* translation initiation codon varies and range between 11 and 75 nucleotides (Figure S2b). Within these intergenic regions, we identified Shine–Dalgarno (SD) sequences (*e.g*., 5’−GGAGG) for some mycobacteria. In *M. terrae*, the intergenic region is 11 nucleotides long and the putative SD (5’−AGGAGG) is located one base downstream of the tRNA^Sec^ 3’ CCA sequence. For other mycobacteria, we could not identify any obvious ribosomal binding site (*i.e*., SD) close to the predicted *selA* translation initiation codon, *e.g*. *M. marinum* where the intergenic region is 14 nucleotides long (Figures 3 and S2b). The absence of SD sequence suggests alternative(s) to initiate translation of the *selAB* mRNA in mycobacteria lacking the SD sequence (see also below) [77].

## Identification of Selenocysteine insertion sequence (SECIS) elements and selenoprotein genes

**Figure 4.**
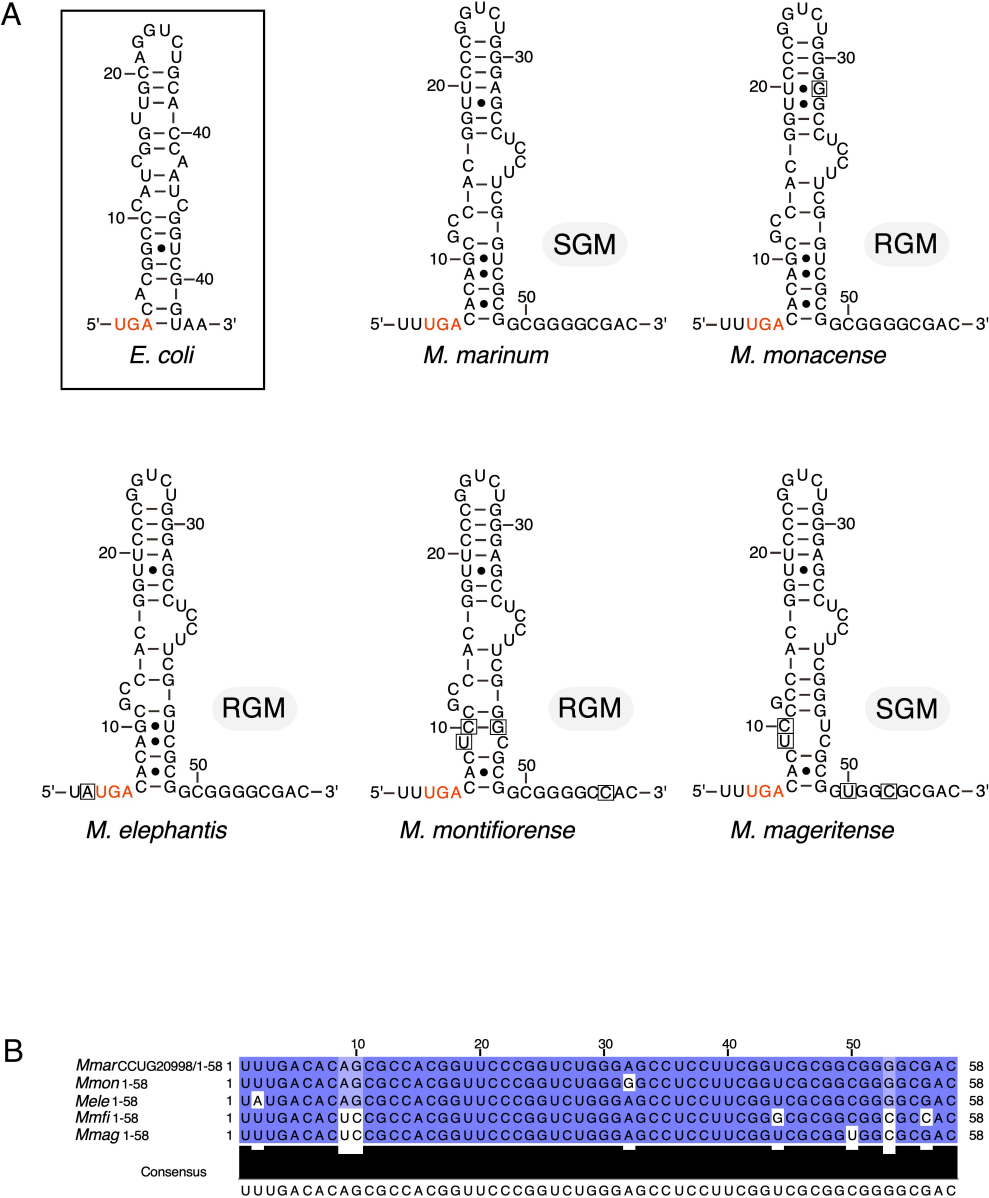
Selenocysteine insertion sequence (SECIS) element. (a) Predicted secondary structures for *E. coli* and indicated mycobacteria. The required UGA codons are marked in red and box nucleotides refer to differences for the displayed mycobacteria relative to the *M. marinum* SECIS element. (b) Sequence alignment of the SGM and RGM shown in (a).

The selenocysteine insertion sequence, SECIS, is a signature for incorporation of Sec, and SECIS together with the UGA codon are essential to insert selenocysteine into selenoproteins (see above). We used the Rfam database along with the infernal cmsearch tool [14,30,78] and identified SECIS elements in 54 mycobacterial genomes. Moreover, based on a homolog based blastN search [50], we identified SECIS element in 105 genomes (Figure 1 and Table S2a). Following this, we randomly verified some of the SECIS element hits using the online bSECISearch tool [31], which confirmed the predicted sequences as SECIS homologs. The combined Rfam and blastN search data suggested one SECIS element per mycobacterial genome. However, searching the 244 genomes using the selenoprofile tool (see above) and blastN homology we predicted the presence of SECIS in 92 mycobacterial genomes. This discrepancy is likely due to gene truncation (*e.g*., in *M. ulcerans* and *M. liflandii*; discussed above, see also below) resulting in pseudogenes (Table S2). Prediction of the secondary SECIS structures for a selected number of mycobacteria revealed similar stem-loop structures with bulges separating the base-paired regions (Figure 4; for alignment of mycobacterial SECIS sequences, Figure S3). The mycobacterial SECIS secondary structures show similarities to other bacterial SECIS structures, for example those present in *E. coli* (Figure 4(a)) [79].

The SECIS element is located immediately 3’ of the UGA in *fdhA*, which is annotated as the formate dehydrogenase subunit α precursor gene (Figures 3 and S2a; see also Table S2). The *fdhA* UGA codon is in frame with the annotated initiation codon of *fdoG*, which is annotated as the formate dehydrogenase-O major subunit precursor gene. Translation of the ‘*fdhA-fdoG*’ transcript (and insertion of Sec) would generate the selenoprotein FDH-O (formate dehydrogenase-O) with a length of 1064 amino acids in *M. marinum*. Comparing *E. coli* and *M. marinum* FDH-O selenoproteins show 38.7% identity (with 95% query coverage) and similar length, 1015 *vs*. 1064 amino acids. The majority of mycobacteria have *fdoG*. However, some mycobacteria have *fdnG* and *fdhA*_1M or *fdhA*_2M in frame with the formate dehydrogenase subunit α precursor gene (*fdhA*). These annotated genes encode for formate dehydrogenase nitrate-inducible and formate dehydrogenase major precursors, respectively (M refers to major and annotation of *fdhA*_1M or *fdhA*_2M, which depends on gene synteny relative to *fdhA*; Table S2b) [76,80]. Together, this would be consistent with that these genes have different origins and horizontally transferred to the respective mycobacteria (see below). Noteworthy, *E. coli* FDH-O is produced both under aerobic and anaerobic conditions and oxidize formate to CO_2_ but it can also catalyse the reverse reaction [81]. Mycobacteria are aerobic bacteria but they survive under oxygen depletion, *e.g*. within macrophages in granulomas [82]. This raises the question whether mycobacteria use formate under limited oxygen conditions.

We conclude that mycobacteria (94 of the 244 mycobacterial genomes; Figure 1) encode for a single selenoprotein, the formate dehydrogenase-O protein, FDH-O, in the majority of mycobacteria. Furthermore, the majority of FDHs in prokaryotes, *e.g*. *E. coli* FDH selenoproteins, rely on molybdenum for activity and hence this would suggest that this is also the case for mycobacterial FDHs [80,83].

Together, these and the data discussed above suggest that roughly 40% of the 244 mycobacterial genomes contain the Sec-machinery, *selA*, *selB*, *selD*, *selC* (tRNA^Sec^) and one single selenoprotein, hereafter referred to as FDH, as revealed by the presence of SECIS in the corresponding mRNA (Figure 1 and Table S2). Interestingly, for some mycobacteria we detected the presence of FDH pseudogenes. In particular noteworthy, while *M. ulcerans*^Agy99^, *M. pseudoshottsii*^DSM45108^ and *M. liflandii*^128FXT^ were predicted to have SECIS these mycobacteria carry FDH pseudogenes, likely due to genome reduction during their evolution [26,27,72,74,75].

## The mycobacterial Sec-machinery and FDH are likely acquired through horizontal gene transfer

The Sec-machinery, tRNA^Sec^ and FDH genes were predicted to be absent in several mycobacterial genomes, and among these, in members of the earliest mycobacterial lineage (see above; Figure 1). In addition, gene syntenies suggested that for several mycobacteria carrying these genes, genes with hypothetical functions are located in close proximity of *selC* (Figures 3 and S2a). Together this raises the question whether the Sec-machinery (including tRNA^Sec^) and FDH genes were acquired during the evolution of the *Mycobacterium* genus. For some, however, these genes have been lost after acquisition due to reduction of their genomes. This is exemplified by the analysis of members of the *M. ulcerans* clade (see above; Figure 1). Hence, we generated phylogenetic trees for the individual Sec-machinery and FDH genes to provide further insight and to understand their interrelation.

For *selA* and *selB*, which are transcribed in the same orientation as *selC*, the phylogeny suggest that these genes are grouped in two main clusters, 1 and 2. Cluster 2 can be subdivided into two subclusters, referred to as 2.1 and 2.2 (Figure S4a, b). Mycobacteria belonging to the same clade group together with the exception for members of the *M. gordonae* clade, which group to both clusters 1 and 2. The analysis also revealed that the main clusters (1 and 2) include both SGM and RGM (*i.e*., slow and rapid growing mycobacteria, respectively, see above).

Albeit the short sequence, the phylogeny for *selC* suggested in principle three clusters with two clusters encompassing SGM and one RGM (as for *selA* and *selB*). For *selC*, however, RGM and SGM are separated except for *M. mageritense* clade members (RGM), which are grouped in cluster 1 among SGM (Figure S4c). Also, the *M. gordonae* clade members are separated where *M. gordonae* and *M. asiaticum* belong to cluster 1 and *M. kubicae*, *M. intermedium* and *M. bourgelatii* to cluster 2.

The phylogeny for *selD*, which is transcribed in the opposite direction relative to *selA*, *selB* and *selC* (see above), showed a similar pattern as the *selA* and *selB* phylogeny (cf. Figure S4a-d). For FDH, the phylogeny also suggested separation into two main clusters. But, here cluster 1 and 2 encompassed mainly RGM and SGM, respectively, with a few exceptions (Figure S4e). For example, *M. marinum*, *M. kansasii* and *M. doricum* group together with RGM in cluster 1, and *M. mageritense* clade members among SGM in cluster 2.1. In this context, *M. mageritense* clade members encode the formate dehydrogenase nitrate-inducible variant, FDH-G (see above; Table S2b).

These data, together with the gene synteny (Figures 3 and S2a), suggest that the Sec-machinery, tRNA^Sec^ and FDH genes have been acquired (and lost) during the evolution of the *Mycobacterium* genus.

## Transcription of Sec-machinery and FDH genes

Having identified the presence of the Sec-machinery genes and the selenoprotein FDH genes among mycobacteria, we were interested in investigating the transcript levels of these genes (if they are transcribed) in exponential and stationary cells, and in response to exposure to different stress conditions. Hence, we analysed RNA-Seq data for the *M. marinum* strain CCUG20998 (*Mmar*^CCUG^) grown under different conditions (see Materials and Methods) [37,38]. In this context, we also analysed the transcription profiles of the same genes for the SGM *M. montifiorense*^DSM44602^ (*Mmfi*) and the three RGM, *M. monacense*^DSM44395^ (*Mmon*^pD5^), *M. elephantis*^DSM44368^ (*Mele*; as discussed above a hypothetical gene is located between *selC* and *selA* in this species) and *M. mageritense*^DSM44476^ (*Mmag*). The RNA-Seq mRNA data for these mycobacteria (*Mmfi*, *Mmon*^pD5^, *Mele* and *Mmag*) were generated using total RNA isolated from exponential growing cells and stationary cells (see Materials and Methods). The SigE mRNA levels were used as a reference.

**Figure 5.**
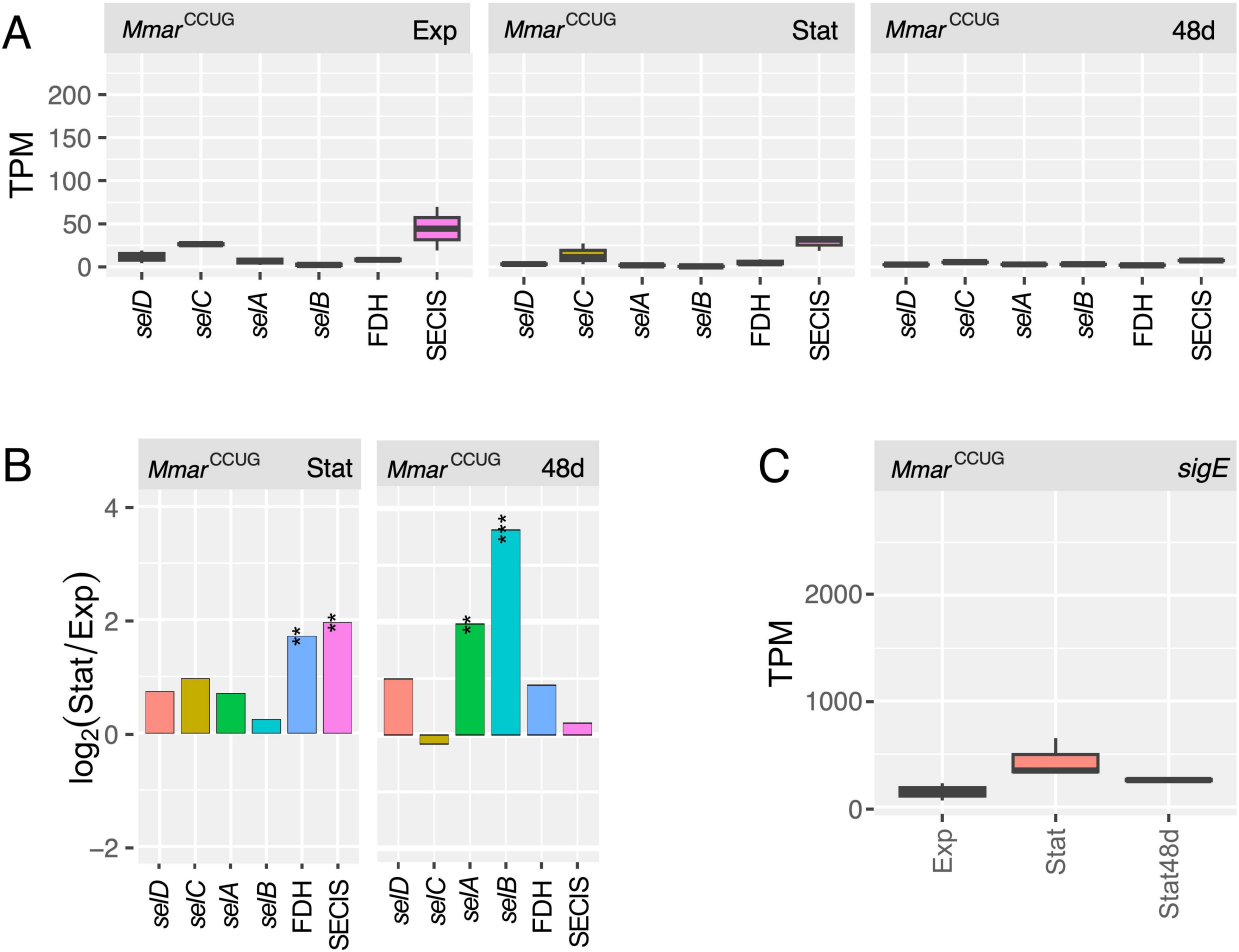
Transcript levels of *M. marinum* CCUG Sec machinery genes and *sigE*.

The mRNA levels were calculated such that, distribution corresponds to levels expressed as transcription per kilobase million values (TPM) [84] and change corresponds to mRNA levels comparing exponential and stationary growth phase. In addition, we analysed changes in mRNA levels in response to exposure to different stressors relative to the control. Some of the stressors are relevant to mycobacterial infection, *e.g*., acidic, exposure to the antibiotic isoniazid and microaerobic stress (see also [37,38]). The data are shown in Figures 5 and S5. Notable, variation in the transcript levels might either be due to change in expression and/or the result of differences in processing or degradation.

Distribution of Sec-machinery, FDH and tRNA^Sec^ transcript levels in exponential, stationary and 48 days old *Mmar*^CCUG^ cells shown as TPM values (see Materials and Methods). Change in Sec-machinery and FDH mRNA levels, and tRNA^Sec^ levels, comparing levels in exponentially growing cells with stationary and 48 days old *Mmar*^CCUG^ cells. The values are expressed in log_2_-fold change as indicated. See main text for details and Materials and Methods. Statistical significance: *p+adj < 0.05; **: p+adj < 0.01; ***: gene among the mycobacterial genomes matches the presence of+adj < 0.001. TPM values for *sigE* mRNA levels in exponential, stationary and 48 days old *Mmar*^CCUG^ cells.

For *Mmar*^CCUG^, the Sec-machinery (including tRNA^Sec^) and FDH transcript levels were low relative to *sigE* mRNA. This was observed both in exponential and stationary cells (Figure 5(a,c); with respect to mRNA levels for *sigE* and other sigma factors in *Mmar*^CCUG^ [37,38]). When we compared transcript levels in exponential and stationary *Mmar*^CCUG^ cells we detected almost a two-fold (log_2_-fold) increase in the FDH transcript while for the other transcripts the change was small with no change for *selB* mRNA (Figure 5(b)). However, in late stationary (48 days of growth on 7H10 solid media) *Mmar*^CCUG^ cells we detected larger changes in *selA* and *selB* mRNA levels while no difference was detected for tRNA^Sec^ (*selC*) relative to exponentially growing cells (cf. *Mmar*^CCUG^ Figure 5(a,b)). For the FDH mRNA (48 days), the level was also slightly increased but less increase was detected than comparing exponential and ‘early’ stationary cells. Similar transcript level patterns as for *Mmar*^CCUG^ were also detected in three other *M. marinum* strains [27], *Mmar*^1218R^, *Mmar*^1218S^ and *Mmar*^M^ (Figure S5). We also detected differences in FDH and SECIS transcript levels, which might be related to processing/degradation of the corresponding transcripts. Moreover, we also detected increases in Sec-machinery mRNA (except for *selD*) and tRNA^Sec^ levels upon exposure of *Mmar*^CCUG^ to starvation (Figure S6). Exposure to other stress conditions such as heat resulted in increased transcript levels of *selA* and tRNA^Sec^ (for other stress conditions see Figure S6 and below).

We conclude that the Sec-machinery and FDH genes are expressed in *M. marinum* with higher levels in stationary cells and under certain stress conditions. Although, the mRNA levels were low in both exponential and stationary cells compared to SigE mRNA levels. The low levels might be related to the presence of only one selenoprotein in mycobacteria.

To understand whether the transcript levels is similar in other mycobacteria, we studied RNA-Seq data of total RNA isolated from exponential and stationary *Mmfi*, *Mmon*^pD5^, *Mele* and *Mmag* cells (see above). The data are shown in Figure S7. As for *M. marinum*, the TPM values suggest that the transcript levels for *Mmon*^pD5^ (Figure S7a-c), *Mele* (Figure S7d-f), and *Mmfi* and *Mmag* (Figure S7i-h) were low in both exponential and stationary cells (cf. Sec-machinery, FDH and SigE mRNA levels). In contrast to *M. marinum*, the Sec-machinery mRNA and tRNA^Sec^ levels decreased in stationary *Mmon*^pD5^ cells (14 days old cultures) while no or just small changes were detected in 6 and 48 days old *Mmon*^pD5^ cells (Figure S7c). Similar patterns were also observed in 17 and 31 days old *Mele* cells (Figure S7f), while the changes for *Mmag* was at the most modest (Figure S7h). Moreover, transcript levels in exponential *vs*. stationary cells revealed almost no changes in the transcription profiles for the SGM *Mmfi* except for *selD* mRNA, which showed a modest increase in stationary cells (Figure S7h). Together, it appears that upon ageing, exponential *vs*. stationary cells, the transcript levels of the Sec-machinery and FDH genes differ comparing the SGM *M. marinum* (and *Mmfi*) and RGM, in particular *Mmon*^pD5^ and *Mele*.

## Transcript levels of Sec-machinery and FDH genes, and tRNA^Sec^ during oxygen depletion

**Figure 6.**
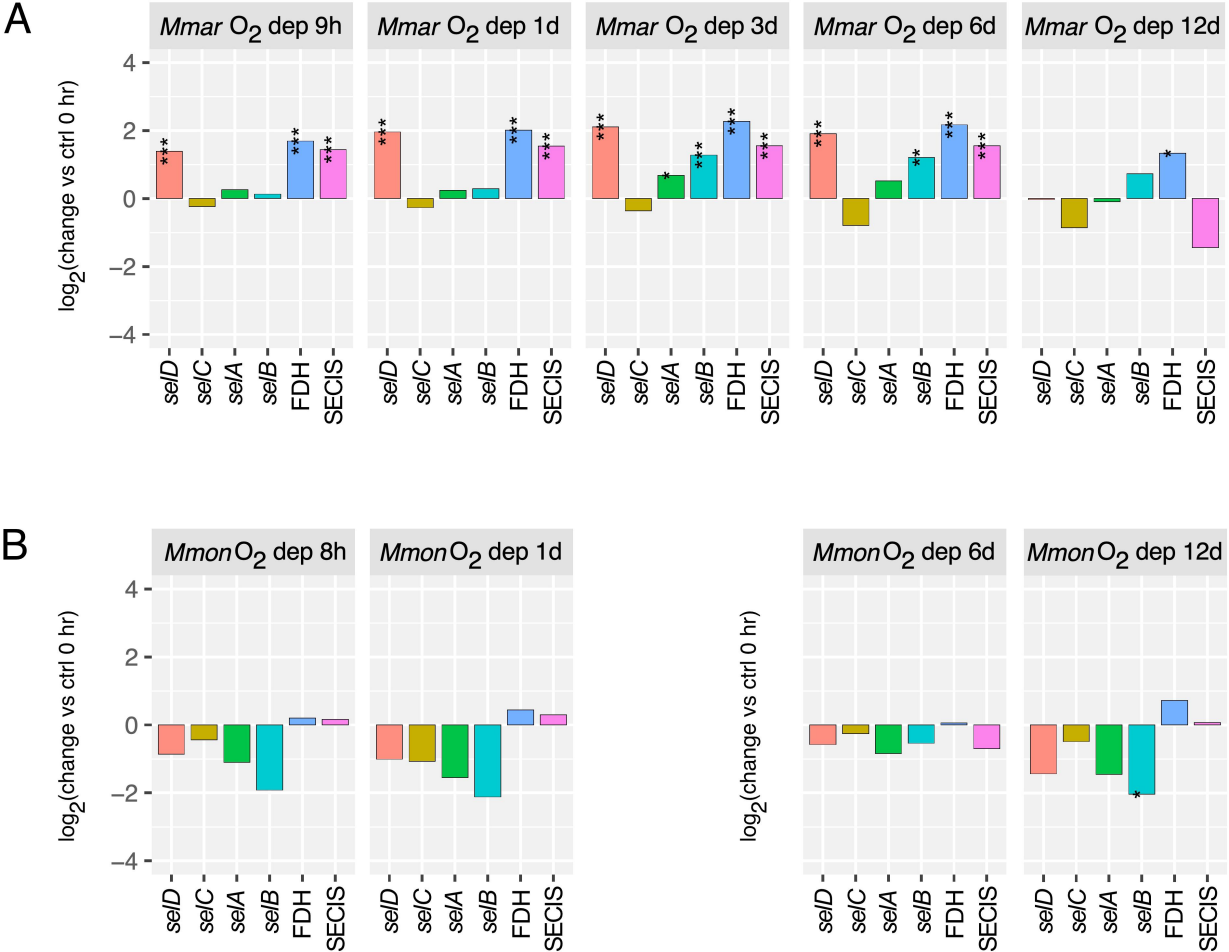
Change in transcript levels of Sec-machinery and FDH genes in *Mmar*^CCUG^ and *Mmon*^pD5^ in response to oxygen depletion (microaerobic stress). (a) *Mmar*^CCUG^, change in *selA*, *selB*, *selD* and FDH mRNA levels, and tRNA^Sec^ (*selC*) and SECIS levels after 9 hrs, 1 day, 4 days, 6 days and 12 days of oxygen depletion relative to the control, i.e. 0 hr. Control corresponds to transcript levels in exponentially growing cells (see Materials and Methods). (b) *Mmon*^pD5^, change in *selA*, *selB*, *selD* and FDH mRNA levels, and tRNA^Sec^ (*selC*) and SECIS levels after 8 hrs, 1 day, 6 days and 12 days of oxygen depletion relative to the control, i.e. 0 hr, see (a).

Mycobacteria are aerobic bacteria but they can adapt to survive under limited oxygen supply, *e.g*., during infection in granulomas [82]. An *in vitro M. tuberculosis* model for oxygen depletion, which mimics the situation in granulomas, was developed by Wayne and co-workers [39,40]. We used this model to investigate the Sec-machinery and FDH mRNA levels as well as levels of tRNA^Sec^ in the SGM *Mmar*^CCUG^ and the RGM *Mmon*^pD5^ in response to oxygen depletion ([37] and Ramesh et al., unpublished). For both mycobacteria, total RNA was extracted at different time points after induction of oxygen depletion and subjected to RNA-Seq (see Materials and Methods). The data are shown in Figure 6(a) (*Mmar*^CCUG^) and Figure 6(b) (*Mmon*^pD5^), see also Figure S8a-d.

The transcript levels are expressed as log_2_-fold. Statistical significance, see Materials and Methods; *p+adj < 0.05; **p+adj < 0.01; ***: p+adj < 0.001.

For *Mmar*^CCUG^, we detected an increase in *selD* and FDH mRNA at the early time points (9 hours and 1 day), while the *selB* mRNA level increases after 3 and 6 days of oxygen depletion. After 12 days, we detected only small variations compared to the control. For tRNA^Sec^, we detected only a small increase over time.

For *Mmon*^pD5^, the response was different. The mRNA levels for the Sec-machinery genes and the tRNA^Sec^ level decreased compared to the control, while no change in the FDH mRNA level was detected. This is similar to the patterns observed in ageing cells (cf. Figure 6(b) and Figure S7c).

Together, these data suggest that Sec-machinery, FDH and tRNA^Sec^ transcript levels differed in response to oxygen depletion comparing the SGM *M. marinum* and the RGM *M. monacense*. Moreover, the higher levels in *M. marinum* in response to oxygen limitation would be in keeping with that it can survive in granulomas in infected fish [85].

### Are *selC* and *selAB* transcribed together?

For the mycobacteria having the tRNA^Sec^ gene (*selC*) located immediately upstream of *selAB* the distance varies between 11 and 75 nucleotides and for *M. marinum* it is 14 nucleotides (see above; Figures 3 and S2). To understand whether *selC* is transcribed together with *selAB*, paired-end read mapping indicated that this is the case for the four *M. marinum* strains (Figure S9a-d). In contrast, no paired-end readings were detected for *M. elephantis*, which possess a gene between *selC* and *selA* (Figure S9e). For *Mmfi*, *Mmon* and *Mmag* where *selC* is positioned immediately upstream of *selA* (similar to *Mmar*), pair-end read mapping also suggested that *selC* and *selA* are transcribed together (Figure S9f-h).

These data suggest that *selC* and *selAB* are transcribed together in *Mmar*, *Mmfi*, *Mmon* and *Mmag* but not in *Mele*.

### A model for the involvement of tRNA^Sec^ in the regulation of *selAB*

**Figure 7.**
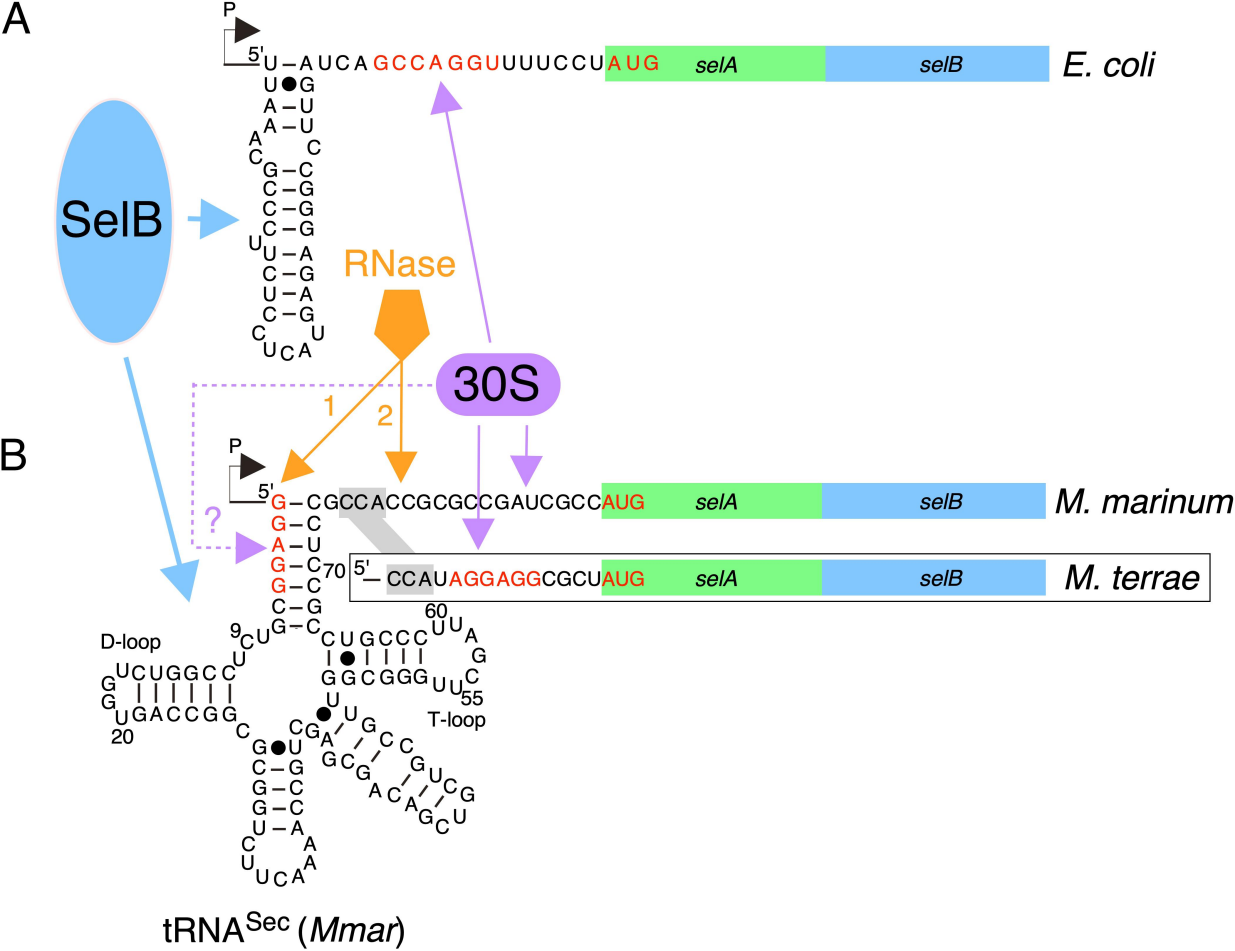
Model of the regulation of *selAB* expression.

In *E. coli*, a SECIS-like element is present in the 5’UTR (untranslated region) of the *selAB* transcript while the tRNA^Sec^ gene (*selC*) is located elsewhere on the chromosome. The distance between the SECIS-like element and the SelA Shine–Dalagarno (SD) sequence is only three nucleotides (Figure 7(a)). This element is recognized by SelB and when it is in complex with GTP and Sec-tRNA^Sec^ it binds to the SECIS-like structure thereby represses *selAB* expression [79]. In mycobacteria, no SECIS-like structure could be identified in this region, instead *selC* is present upstream of *selAB*. For several mycobacteria, including *M. marinum*, no apparent SD-sequence is present upstream of the predicted SelA initiation codon while other mycobacteria have an SD-sequence (Figure S2b). In the most extreme case, *M. terrae*, the distance between *selC* and the predicted SelA translation initiation codon is 11 nucleotides, which includes an SD-sequence beginning one nucleotide downstream of the 3’ CCA end of tRNA^Sec^ (Figure 7(b)). Our data further suggested that the tRNA^Sec^ gene and *selA* are transcribed together in selected mycobacteria (*Mmar*, *Mmfi*, *Mmon* and *Mmag*; Figure S8). Combined, these findings raise the question whether the tRNA^Sec^ gene or the native unprocessed/processed tRNA^Sec^ transcript is involved in the regulation of *selAB* expression. One possibility is that SelB binds to the native unprocessed tRNA^Sec^ transcript thereby affecting the expression of *selC*, *selA* and *selB*. Compared to *E. coli* this would indicate a similar way to regulate the expression of *selA* and *selB*, except that in mycobacteria this would involve the transcription of *selC* (Figure 7(b)). Binding of SelB to the unprocessed tRNA^Sec^ transcript might also influence processing and thereby the level of functional tRNA^Sec^. We can also envision that during transcription the GGAGG-sequence in the tRNA^Sec^ acceptor-stem acts as an SD-sequence (consensus 5’−AGAAAGGAGG) interacts with the 30S ribosomal subunit thereby preventing folding and processing of tRNA^Sec^. As a consequence, this would promote interaction with the *selA* SD-sequence (when present). Translational initiation in mycobacteria lacking a *selA* SD-sequence might be achieved through alternative routs [77] and/or involving the GGAGG-sequence in the tRNA^Sec^ acceptor-stem. We also note that 6C RNA acts as regulator targeting, via its C-rich loops, different mRNAs in mycobacteria [86]. Our preliminary data suggest that the SelA mRNA level is affected by the level of 6C RNA in *M. marinum*. It is therefore conceivable that the C-rich 6C RNA binds to the tRNA^Sec^ 5’ G_−1_GGAGGCG-sequence (Figures 2 and S1) during transcription and thereby affect the tRNA^Sec^ and SelAB mRNA levels. Moreover, in *E. coli* the endoribonuclease RNase P cleaves pre-tRNA transcripts with more than one tRNA in 3’ to 5’ direction [87,88]. We can therefore not exclude that the ‘*selC-selA-selB*’ transcript is first processed by RNase P and other ribonucleases resulting in separation of the tRNA^Sec^ and *selAB* mRNA. Regardless of the way the expression of *selAB* is regulated it is clear from our data that the levels of the Sec-machinery gene transcripts are low and this might be the result of coupling between transcription, tRNA processing and translation (and the presence of only one selenoprotein among mycobacteria, see above). In this context, we note that the folding of the 5’ UTR (operator) of the *E. coli* threonyl-tRNA-synthetase (ThrRS) mRNA is structural similar to tRNA^Thr^. The operator interacts with ThrRS and thereby represses the expression of ThrRS and degradation of the mRNA [89].

In *E. coli* SelB in complex with Sec-tRNA^Sec^ and GTP (in blue) interacts with the SECIS-like element located upstream of the *selAB* ribosomal binding site (Shine–Dalgarno, SD, sequence marked in red and the ribosomal 30S subunit in purple) resulting in repression of translation of SelAB (see main text and [79]). The black arrow indicates the transcription start, P. The location of the *M. marinum* tRNA^Sec^ upstream of *selAB*, upper sequence, and absence of a ribosomal binding site, SD. The lower boxed sequence refers to the sequence present in *M. terrae*, which includes a ribosomal binding site, SD, marked in red. Residues G1 to G5 in the acceptor stem are marked in red and the 3’ CCA residues are highlighted in grey. The putative interaction between SelB (in blue) and tRNA^Sec^ is indicated with a blue arrow. The orange marked RNase 1 and 2 arrows correspond to RNases involved in trimming of the tRNA^Sec^ 5’ (RNase P) and 3’ ends. As in (a) the purple 30S ribosomal subunit binds to the SD in *M. terrae* and inititate translation starting at the red marked AUG codon. In *M.* *marinum* translation initiation of SelAB proceeds by a different mechanism operating on mRNAs lacking SD [77]. Irrespective of binding of the ribosome upstream of the SelAB AUG, the 30S subunit might bind to the tRNA^Sec^ G1-G5 sequence (which corresponds to an SD sequence) during transcription prior to folding of tRNA^Sec^ (marked by the dashed purple arrow) and thereby affect the translation of SelAB. See also the main text.

To conclude, the way the expression of *selC*, *selA* and *selB* are regulated in mycobacteria differs compare to *E. coli*. The regulation of these genes remains to be studied to understand whether the regulation occurs at the transcription and/or the translational levels.

## Conclusion

We provide data that among 244 mycobacterial genomes, approximately 40% encode for tRNA^Sec^ (*selC*), the Sec-machinery and the predicted sole selenoprotein FDH with its relatively well-conserved SECIS element. Mycobacteria are aerobic but survives under anoxic conditions. It is therefore conceivable that FDH in mycobacteria are needed for survival under conditions where oxygen is limited. The genes appear to have been acquired through horizontal gene transfer during the evolution of the genus. For some, as exemplified by *M. ulcerans* clade members, these genes have been lost due to genome reduction. Furthermore, dependent on mycobacteria the origin of the genes seems to differ. Our data also suggest a tRNA^Sec^ variant present in mycobacteria with its 7 bp amino acid acceptor-stem and 6 bp T-stem, which nevertheless comply to the common tRNA^Sec^ 13 bp ‘acceptor-T-stem’ domain. Our present findings establish the groundwork to develop experimental protocols to address, *e.g*. the evolution and regulation of the mycobacterial Sec-machinery and the predicted sole selenoprotein FDH. Finally, in the context of that inorganic Se compounds are metabolized in some Se-enriched bacteria (see *e.g* [90], and references therein) an interesting question would be to understand if this also applies to mycobacteria and how (and if) addition of selenium affect mycobacterial growth.

## Supplementary Material

Ms Behra et al SUPP INFO.pdf

Ms Behra et al SUPPLEMENTARY INFORMATION FILE TABLES S1 S3 S3.xlsx

## Data Availability

The RNA-seq datasets for *M. montefiorense*, *M. elephantis* and *M. mageritense* generated in this study are available under BioProject PRJNA1468449. All RNA-seq datasets used in this study are listed in Table S1b. Additional details are available from the corresponding author upon reasonable request.

## References

[1] Flohé L. The labour pains of biochemical selenology: the history of selenoprotein biosynthesis. Biochim Biophys Acta. 2009;1790(11):1389–1403. doi: 10.1016/j.bbagen.2009.03.031 19358874

[2] Rayman MP. Selenium and human health. Lancet Lond Engl. 2012;379(9822):1256–1268. doi: 10.1016/S0140-6736(11)61452-9 22381456

[3] Böck A, Forchhammer K, Heider J, et al. Selenocysteine: the 21st amino acid. Mol Microbiol. 1991;5(3):515–520. doi: 10.1111/j.1365-2958.1991.tb00722.x 1828528

[4] Rother M. Prokaryotic selenoprotein biosynthesis and function. In: Hatfield DL, Schweizer U, Tsuji PA Gladyshev VN, editors. Selenium: its molecular biology and role in human health. Cham: Springer International Publishing; 2012. p. 47–58.

[5] Zinoni F, Heider J, Böck A. Features of the formate dehydrogenase mRNA necessary for decoding of the UGA codon as selenocysteine. Proc Natl Acad Sci USA. 1990;87(12):4660–4664. doi: 10.1073/pnas.87.12.4660 2141170 PMC54176

[6] Hüttenhofer A, Westhof E, Böck A. Solution structure of mRNA hairpins promoting selenocysteine incorporation in *Escherichia coli* and their base-specific interaction with special elongation factor SELB. RNA. 1996;2(4):354–366. 8634916 PMC1369378

[7] Leinfelder W, Zehelein E, Mandrand-Berthelot M, et al. Gene for a novel tRNA species that accepts L-serine and cotranslationally inserts selenocysteine. Nature. 1988;331(6158):723–725. doi: 10.1038/331723a0 2963963

[8] Yoshizawa S, Böck A. The many levels of control on bacterial selenoprotein synthesis. Biochim Biophys Acta. 2009;1790(11):1404–1414. doi: 10.1016/j.bbagen.2009.03.010 19328835

[9] Wells M, Stolz JF. Microbial selenium metabolism: a brief history, biogeochemistry and ecophysiology. FEMS Microbiol Eco. 2020;96(12):fiaa209. doi: 10.1093/femsec/fiaa209 33045045

[10] Leinfelder W, Forchhammer K, Zinoni F, et al. *Escherichia coli* genes whose products are involved in selenium metabolism. J Bacteriol. 1988;170(2):540–546. doi: 10.1128/jb.170.2.540-546.1988 2962989 PMC210687

[11] Korkmaz G, Holm M, Wiens T, et al. Comprehensive analysis of stop codon usage in bacteria and its correlation with release factor abundance. J Biol Chem. 2014;289(44):30334–30342. doi: 10.1074/jbc.M114.606632 25217634 PMC4215218

[12] Gursinsky T, Jäger J, Andreesen JR, et al. A *selDABC* cluster for selenocysteine incorporation in *Eubacterium acidaminophilum*. Arch Microbiol. 2000;174(3):200–212. doi: 10.1007/s002030000196 11041351

[13] Kryukov GV, Gladyshev VN. The prokaryotic selenoproteome. EMBO Rep. 2004;5(5):538–543. doi: 10.1038/sj.embor.7400126 15105824 PMC1299047

[14] Zhang Y, Fomenko DE, Gladyshev VN. The microbial selenoproteome of the Sargasso Sea. Genome Biol. 2005;6(4):R37. doi: 10.1186/gb-2005-6-4-r37 15833124 PMC1088965

[15] Zhang Y, Romero H, Salinas G, et al. Dynamic evolution of selenocysteine utilization in bacteria: a balance between selenoprotein loss and evolution of selenocysteine from redox active cysteine residues. Genome Biol. 2006;7(10):R94. doi: 10.1186/gb-2006-7-10-r94 17054778 PMC1794560

[16] Zhang Y, Gladyshev VN. Trends in selenium utilization in Marine microbial world revealed through the analysis of the global ocean sampling (GOS) project. PLoS Genet. 2008;4(6):e1000095. doi: 10.1371/journal.pgen.1000095 18551170 PMC2398784

[17] Das S, Pettersson BMF, Behra PRK, et al. Characterization of three *Mycobacterium* spp. With potential use in bioremediation by genome sequencing and Comparative genomics. Genome Biol Evol. 2015;7(7):1871–1886. doi: 10.1093/gbe/evv111 26079817 PMC4524478

[18] Lin J, Peng T, Jiang L, et al. Comparative genomics reveals New Candidate genes involved in selenium metabolism in prokaryotes. Genome Biol Evol. 2015;7(3):664–676. doi: 10.1093/gbe/evv022 25638258 PMC5322559

[19] Mariotti M, Santesmasses D, Capella-Gutierrez S, et al. Evolution of selenophosphate synthetases: emergence and relocation of function through independent duplications and recurrent subfunctionalization. Genome Res. 2015;25(9):1256–1267. doi: 10.1101/gr.190538.115 26194102 PMC4561486

[20] Mukai T, Englert M, Tripp HJ, et al. Facile recoding of selenocysteine in Nature. Angew Chem Int Ed. 2016;55(17):5337–5341. doi: 10.1002/anie.201511657 PMC4833512 26991476

[21] Peng T, Lin J, Xu Y-Z, et al. Comparative genomics reveals new evolutionary and ecological patterns of selenium utilization in bacteria. Isme J. 2016;10(8):2048–2059. doi: 10.1038/ismej.2015.246 26800233 PMC5029168

[22] Santesmasses D, Mariotti M, Guigó R. Computational identification of the selenocysteine tRNA (tRNASec) in genomes. PLOS Comput Biol. 2017;13(2):e1005383. doi: 10.1371/journal.pcbi.1005383 28192430 PMC5330540

[23] Santesmasses D, Mariotti M, Guigó R. Selenoprofiles: a Computational pipeline for annotation of selenoproteins. In: Chavatte L, editor. Selenoproteins: methods and protocols, methods in molecular biology. New York (NY): Springer; 2018. p. 17–28. 10.1007/978-1-4939-7258-6_2 28917034

[24] Santesmasses D, Mariotti M, Gladyshev VN. Bioinformatics of Selenoproteins. Antioxid Redox Signal. 2020;33(7):525–536. doi: 10.1089/ars.2020.8044 32031018 PMC7409585

[25] Farukh M. Comparative genomic analysis of selenium utilization traits in different marine environments. J Microbiol. 2020;58(2):113–122. doi: 10.1007/s12275-020-9250-0 31993987

[26] Behra PRK, Pettersson BMF, Ramesh M, et al. Comparative genome analysis of mycobacteria focusing on tRNA and non-coding RNA. BMC Genomics. 2022;23(1):704. doi: 10.1186/s12864-022-08927-5 36243697 PMC9569102

[27] Das S, Pettersson BMF, Behra PRK, et al. Extensive genomic diversity among *Mycobacterium marinum* strains revealed by whole genome sequencing. Sci Rep. 2018;8(1):12040. doi: 10.1038/s41598-018-30152-y 30104693 PMC6089878

[28] Lowe TM, Eddy SR. tRNAscan: a program for improved detection of transfer RNA genes in genomic sequence. Nucl Acids Res. 1997;25(5):955–964. doi: 10.1093/nar/25.5.955 9023104 PMC146525

[29] Chan PP, Lowe TM. tRNAscan: searching for tRNA genes in genomic sequences. Methods Mol Biol. 2019;1962:1–14. 31020551 10.1007/978-1-4939-9173-0_1 PMC6768409

[30] Kalvari I, Argasinska J, Quinones-Olvera N, et al. Rfam 13.0: shifting to a genome-centric resource for non-coding RNA families. Nucl Acids Res. 2018;46(D1):D335–D342. doi: 10.1093/nar/gkx1038 29112718 PMC5753348

[31] Zhang Y, Gladyshev VN. An algorithm for identification of bacterial selenocysteine insertion sequence elements and selenoprotein genes. Bioinformatics. 2005;21(11):2580–2589. doi: 10.1093/bioinformatics/bti400 15797911

[32] Larkin MA, Blackshields G, Brown NP, et al. Clustal W and clustal X version 2.0. Bioinformatics. 2007;23(21):2947–2948. doi: 10.1093/bioinformatics/btm404 17846036

[33] Waterhouse AM, Procter JB, Martin DMA, et al. Jalview version 2 - a multiple sequence alignment editor and analysis workbench. Bioinformatics. 2009;25(9):1189–1191. doi: 10.1093/bioinformatics/btp033 19151095 PMC2672624

[34] Katoh K, Standley DM. MAFFT multiple sequence alignment software version 7: improvements in performance and usability. Mol Biol Evol. 2013;30(4):772–780. doi: 10.1093/molbev/mst010 23329690 PMC3603318

[35] Price MN, Dehal PS, Arkin AP. FastTree 2 - approximately maximum-likelihood trees for large alignments. PLOS ONE. 2010;5(3):e9490. doi: 10.1371/journal.pone.0009490 20224823 PMC2835736

[36] Letunic I, Bork P. Interactive tree of life (iTOL) v4: recent updates and new developments. Nucl Acids Res. 2019;47(W1):W256–W259. doi: 10.1093/nar/gkz239 30931475 PMC6602468

[37] Pettersson BMF, Das S, Behra PRK, et al. Comparative sigma factor-mRNA levels in *Mycobacterium marinum* under stress conditions and during Host infection. PLOS ONE. 2015;10(10):e0139823. doi: 10.1371/journal.pone.0139823 26445268 PMC4596819

[38] Behra PRK, Pettersson BMF, Ramesh M, et al. Insight into the biology of *Mycobacterium mucogenicum* and *Mycobacterium neoaurum* clade members. Sci Rep. 2019;9(1):19259. doi: 10.1038/s41598-019-55464-5 31848383 PMC6917791

[39] Wayne LG, Lin KY. Glyoxylate metabolism and adaptation of *Mycobacterium tuberculosis* to survival under anaerobic conditions. Infect Immun. 1982;37(3):1042–1049. doi: 10.1128/iai.37.3.1042-1049.1982 6813266 PMC347645

[40] Wayne LG, Hayes LG. An *in vitro* model for sequential study of shiftdown of *Mycobacterium tuberculosis* through two stages of nonreplicating persistence. Infect Immun. 1996;64(6):2062–2069.39. doi: 10.1128/iai.64.6.2062-2069.1996 8675308 PMC174037

[41] Kim D, Pertea G, Trapnell C, et al. TopHat2: accurate alignment of transcriptomes in the presence of insertions, deletions and gene fusions. Genome Biol. 2013;14(4):R36. doi: 10.1186/gb-2013-14-4-r36 23618408 PMC4053844

[42] Langmead B, Salzberg SL. Fast gapped-read alignment with bowtie 2. Nat Methods. 2012;9(4):357–359. doi: 10.1038/nmeth.1923 22388286 PMC3322381

[43] Anders S, Pyl PT, Huber W. Htseq—a python framework to work with high-throughput sequencing data. Bioinformatics. 2015;31(2):166–169. doi: 10.1093/bioinformatics/btu638 25260700 PMC4287950

[44] Love MI, Huber W, Anders S. Moderated estimation of Fold change and dispersion for RNA-seq data with DESeq2. Genome Biol. 2014;15(12):550. doi: 10.1186/s13059-014-0550-8 25516281 PMC4302049

[45] Alvarez RV, Pongor LS, Marino-Ramirez L, et al. Tpmcalulator: one-step software to quantify mRNA abundance of genomic features. Bioinformatics. 2019;35(11):1960–1962. doi: 10.1093/bioinformatics/bty896 30379987 PMC6546121

[46] R development core team. R: a language and environment for statisticalcomputing. Vienna. Available from: https://www.R-project.org/2008

[47] Wickham H. ggplot2: elegant graphics for data analysis. 2nd ed. Cham, Switzerland: Use R! Springer International Publishing; 2016.

[48] Guy L, Roat Kultima J, Andersson SGE. genoPlotr: comparative gene and genome visualization in R. Bioinformatics. 2010;26(18):2334–2335. doi: 10.1093/bioinformatics/btq413 20624783 PMC2935412

[49] Sweeney BA, Hoksza D, Nawrocki EP, et al. R2DT is a framework for predicting and visualising RNA secondary structure using templates. Nat Commun. 2021;12(1):3494. doi: 10.1038/s41467-021-23555-5 34108470 PMC8190129

[50] Boratyn GM, Camacho C, Cooper PS, et al. BLAST: a more efficient report with usability improvements. Nucl Acids Res. 2013;41(W1):W29–W33. doi: 10.1093/nar/gkt282 23609542 PMC3692093

[51] Cai M, Chen W-M, Nic Y, et al. Complete genome sequence of *Amycolicicocus subflavus*DQS3-9A1 T, an actinomycete isolated from crude oil-polluted soil. J Bacteriol. 2011;193(17):4538–4539. doi: 10.1128/JB.05388-11 21725023 PMC3165522

[52] Hamada M, Shibata C, Sakurai K, et al. Reclassification of *Amycolicicoccus subflavus* as *Hoyosella subflava* comb. nov. And emended descriptions of the *Hoyosella* and *Hoyosella altamirensis*. Int J Syst Evol Microbiol. 2016;66(11):4711–4715. doi: 10.1099/ijsem.0.001415 27514929

[53] Baron C, Böck A. The length of the aminoacyl-acceptor stem of the selenocysteine-specific tRNA(Sec) of *Escherichia coli* is the determinant for binding to elongation factors SELB or Tu. J Biol Chem. 1991;266(30):20375–20379. doi: 10.1016/S0021-9258(18)54933-4 1939093

[54] Krahn N, Fischer JT, Söll D. Naturally occurring tRNAs with non-canonical structures. Front Microbiol. 2020;11:596914. doi: 10.3389/fmicb.2020.596914 33193279 PMC7609411

[55] Itoh Y, Bröcker MJ, Sekine S, et al. Decameric SelA•tRNASec ring structure reveals mechanism of bacterial selenocysteine formation. Science. 2013;340(6128):75–78. doi: 10.1126/science.1229521 23559248 PMC3976565

[56] Silva IR, Serrao VHG, Manzine LR, et al. Formation of a ternary complex for selenocysteine biosynthesis in bacteria. J Biol Chem. 2015;290(49):29178–29188. doi: 10.1074/jbc.M114.613406 26378233 PMC4705924

[57] Lai LB, Vioque A, Kirsebom LA, et al. Unexpected diversity of RNase P, an ancient tRNA processing enzyme: challenges and prospects. FEBS Lett. 2010;584(2):287–296. doi: 10.1016/j.febslet.2009.11.048 19931535 PMC2799185

[58] Bechhofer DH, Deutscher MP. Bacterial ribonucleases and their roles in RNA metabolism. Crit Rev Biochem Mol Biol. 2019;54(3):242–300. doi: 10.1080/10409238.2019.1651816 31464530 PMC6776250

[59] Burkard U, Söll D. The unusually long amino acid acceptor stem of Escherichia coli selenocysteine tRNA results from abnormal cleavage by RNase P. Nucl Acids Res. 1988;16(24):11617–11624. doi: 10.1093/nar/16.24.11617 3062578 PMC339093

[60] Kirsebom LA, Liu F, McClain WH. The discovery of a catalytic RNA within RNase P and its legacy. J Biol Chem. 2024;300(6):107318. doi: 10.1016/j.jbc.2024.107318 38677513 PMC11143913

[61] Brännvall M, Kikovska E, Wu S, et al. Evidence for induced fit in bacterial RNase P RNA-mediated cleavage. J Mol Biol. 2007;372(5):1149–1154. doi: 10.1016/j.jmb.2007.07.030 17719605

[62] Jackman JE, Gott JM, Gray MW. Doing it in reverse: 3’- to 5’ polymerization by the Thg1 superfamily. RNA. 2012;18(5):886–899. doi: 10.1261/rna.032300.112 22456265 PMC3334698

[63] Das S, Pettersson BMF, Behra PRK, et al. The *Mycobacterium phlei* genome: expectations and surprises. Genome Biol Evol. 2016;8:975–985. doi: 10.1093/gbe/evw049 26941228 PMC4860684

[64] Giegé R, Eriani G. The tRNA identity landscape for aminoacylation and beyond. Nucl Acids Res. 2023;51(4):1528–1570. doi: 10.1093/nar/gkad007 36744444 PMC9976931

[65] Forchhammer K, Leinfelder W, Böck A. Identification of a novel translation factor necessary for the incorporation of selenocysteine into protein. Nature. 1989;342(6248):453–456. doi: 10.1038/342453a0 2531290

[66] Itoh Y, Sekine S, Yokoyama S. Crystal structure of the full-length bacterial selenocysteine-specific elongation factor SelB. Nucl Acids Res. 2015;43(18):9028–9038. doi: 10.1093/nar/gkv833 26304550 PMC4605307

[67] Commans S, Böck A. Selenocysteine inserting tRNAs: an overview. FEMS Microbiol Rev. 1999;23(3):335–351. doi: 10.1111/j.1574-6976.1999.tb00403.x 10371037

[68] Seemann T. Prokka: rapid prokaryotic genome annotation. Bioinformatics. 2014;30(14):2068–2069. doi: 10.1093/bioinformatics/btu153 24642063

[69] Stinear TP, Seemann T, Harrison PF, et al. Insights from the complete genome sequence of *Mycobacterium marinum* on the evolution of *Mycobacterium tuberculosis*. Genome Res. 2008;18(5):729–741. doi: 10.1101/gr.075069.107 18403782 PMC2336800

[70] Tobias NJ, Doig KD, Medema MH, et al. Complete genome sequence of the frog pathogen *Mycobacterium ulcerans* ecovar *liflandii*. J Bacteriol. 2013;195(3):556–564. doi: 10.1128/JB.02132-12 23204453 PMC3554023

[71] Rondini S, Käser M, Stinear T, et al. Ongoing genome reduction in *Mycobacterium ulcerans*. Emerg Inf Dis. 2007;13(7):1008–1015. doi: 10.3201/eid1307.060205 PMC2878211 18214172

[72] Röltgen K, Stinear TP, Pluschke G. The genome, evolution and diversity of *Mycobacterium ulcerans*. Infect Genet Evol. 2012;12(3):522–529. doi: 10.1016/j.meegid.2012.01.018 22306192

[73] Yoshida M, Miyamoto Y, Ogura Y, et al. Complete chromosome sequence of a mycolactone-producing Mycobacterium, *Mycobacterium pseudoshottsii*. Genome Announc. 2017;5(48):e01363–17. doi: 10.1128/genomeA.01363-17 29192083 PMC5722069

[74] Doig KD, Holt KE, Fyfe JAM, et al. On the origin of *Mycobacterium ulcerans*, the causative agent of Buruli ulcer. BMC Genomics. 2012;13(1):258. doi: 10.1186/1471-2164-13-258 22712622 PMC3434033

[75] Röltgen K, Pluschke G. Buruli ulcer: history and disease burden. In: Pluschke G Röltgen K, editors. Buruli Ulcer: *Mycobacterium ulcerans* disease [Internet]. Cham: Springer International Publishing; 2019. p. 1–41.

[76] Blattner FR, G P 3rd, Bloch CA, et al. The complete genome sequence of *Escherichia coli* K-12. Science. 1997;277(5331):1453–1462. doi: 10.1126/science.277.5331.1453 9278503

[77] Sawyer EB, Grabowska AD, Cortes T. Translational regulation in mycobacteria and its implications for pathogenicity. Nucl Acids Res. 2018;46(14):6950–6961. doi: 10.1093/nar/gky574 29947784 PMC6101614

[78] Nawrocki EP, Eddy SR. Infernal 1.1: 100-fold faster RNA homology searches. Bioinformatics. 2013;29(22):2933–2935. doi: 10.1093/bioinformatics/btt509 24008419 PMC3810854

[79] Thanbichler M, Böck A. The function of SECIS RNA in translational control of gene expression in *Escherichia coli*. EMBO J. 2002;21(24):6925–6934. doi: 10.1093/emboj/cdf673 12486013 PMC139081

[80] Hartmann T, Schwanhold N, Leimkühler S. Assembly and catalysis of molybdenum or tungsten-containing formate dehydrogenase from bacteria. Biochim Biophys Acta. 2015;1854(9):1090–1100. doi: 10.1016/j.bbapap.2014.12.006 25514355

[81] Sawers RG. How FocA facilitates fermentation and respiration of formate by *Escherichia coli*. J Bacteriol. 2025;207(2):e0050224. doi: 10.1128/jb.00502-24 39868885 PMC11841067

[82] Kundu M, Basu J. Applications of transcriptomics and proteomics for understanding dormancy and resuscitation in *Mycobacterium tuberculosis*. Front Microbiol. 2021;12:642487. doi: 10.3389/fmicb.2021.642487 33868200 PMC8044303

[83] Nielsen CF, Lange L, Meyer AS. Classification and enzyme kinetics of formate dehydrogenases for biomanufacturing via CO2 utilization. Biotechnol Adv. 2019;37(7):107408. doi: 10.1016/j.biotechadv.2019.06.007 31200015

[84] Zhao S, Ye Z, Stanton R. Misuse of RPKM or TPM normalization when comparing across samples and sequencing protocols. RNA. 2020;26(8):903–909. doi: 10.1261/rna.074922.120 32284352 PMC7373998

[85] Menon AR, Prest RJ, Tobin DM, et al. *Mycobacterium marinum* as a model for understanding principles of mycobacterial pathogenesis. J Bacteriol. 2025;207(5):e0004725. doi: 10.1128/jb.00047-25 40304497 PMC12096832

[86] Mai J, Rao C, Watt J, et al. *Mycobacterium tuberculosis* 6C sRNA binds multiple mRNA targets via C-rich loops independent of RNA chaperones. Nucl Acids Res. 2019;47(8):4292–4307. doi: 10.1093/nar/gkz149 30820540 PMC6486639

[87] Agrawal A, Mohanty BK, Kushner SR. Processing of the seven valine tRNAs in *Escherichia coli* involves novel features of RNase P. Nucl Acids Res. 2014;42(17):11166–11179. doi: 10.1093/nar/gku758 25183518 PMC4176162

[88] Zhao J, Harris ME. Distributive enzyme binding controlled by local RNA context results in 3‘to 5’ directional processing of discistronic tRNA precursors by Escherichia coli ribonuclease P. Nucl Acids Res. 2019;47(3):1451–1467. doi: 10.1093/nar/gky1162 30496557 PMC6379654

[89] Romby P, Springer M. Bacterial translational control at atomic resolution. Trends Genet. 2003;19(3):155–161. doi: 10.1016/S0168-9525(03)00020-9 12615010

[90] Luo L, Hou X, Yi D, et al. Selenium-enriched microoganisms: metabolism, production, and applications. Microorganisms. 2025;13(8):1849. doi: 10.3390/microorganisms13081849 40871353 PMC12388498

